# Seasonality and immunity to laboratory-confirmed seasonal coronaviruses (HCoV-NL63, HCoV-OC43, and HCoV-229E): results from the Flu Watch cohort study

**DOI:** 10.12688/wellcomeopenres.15812.2

**Published:** 2020-12-10

**Authors:** Robert W. Aldridge, Dan Lewer, Sarah Beale, Anne M. Johnson, Maria Zambon, Andrew C. Hayward, Ellen B. Fragaszy, Flu Watch Group

**Affiliations:** 1 UCL Public Health Data Science Research Group, Institute of Health Informatics, UCL, London, NW1 2DA, UK; 2 UCL Research Department of Epidemiology & Public Health, UCL, London, WC1E 7HB, UK; 3 UCL Institute for Global Health, UCL, London, WC1E 6JB, UK; 4 Public Health England, 2-6 Salisbury Square, London, EC4Y 8AE, UK; 5 Department of Infectious Disease Epidemiology, London School of Hygiene and Tropical Medicine, London, WC1E 7HT, UK

**Keywords:** HCoV-NL63, HCoV-OC43, HCoV-229E, SARS-CoV-2, public health, epidemiology, pandemic

## Abstract

**Background: **There is currently a pandemic caused by the novel coronavirus SARS-CoV-2. The intensity and duration of this first and second waves in the UK may be dependent on whether SARS-CoV-2 transmits more effectively in the winter than the summer and the UK Government response is partially built upon the assumption that those infected will develop immunity to reinfection in the short term. In this paper we examine evidence for seasonality and immunity to laboratory-confirmed seasonal coronavirus (HCoV) from a prospective cohort study in England.

**Methods: **In this analysis of the Flu Watch cohort, we examine seasonal trends for PCR-confirmed coronavirus infections (HCoV-NL63, HCoV-OC43, and HCoV-229E) in all participants during winter seasons (2006-2007, 2007-2008, 2008-2009) and during the first wave of the 2009 H1N1 influenza pandemic (May-Sep 2009). We also included data from the pandemic and ‘post-pandemic’ winter seasons (2009-2010 and 2010-2011) to identify individuals with two confirmed HCoV infections and examine evidence for immunity against homologous reinfection.

**Results: **We tested 1,104 swabs taken during respiratory illness and detected HCoV in 199 during the first four seasons. The rate of confirmed HCoV infection across all seasons was 390 (95% CI 338-448) per 100,000 person-weeks; highest in the Nov-Mar 2008/9 season at 674 (95%CI 537-835) per 100,000 person-weeks. The highest rate was in February at 759 (95% CI 580-975) per 100,000 person-weeks. Data collected during May-Sep 2009 showed there was small amounts of ongoing transmission, with four cases detected during this period. Eight participants had two confirmed infections, of which none had the same strain twice.

**Conclusion:** Our results provide evidence that HCoV infection in England is most intense in winter, but that there is a small amount of ongoing transmission during summer periods. We found some evidence of immunity against homologous reinfection.

## Background

We write this paper during a pandemic caused by the novel coronavirus SARS-CoV-2. As of 30 ^th^ November 2020, there were 62,363,527 confirmed cases and 1,456,687 deaths reported ^1^. In the UK, 1,629,657 confirmed cases have been reported and 58,448 patients who tested positive for SARS-CoV-2 died within 28 days of a positive test ^2^. The UK Government aims to reduce the spread of SARS-CoV-2 through mixture social distancing measures including asking people to stay at home and only go outside for food, health reasons or work where this absolutely cannot be done from home. In addition to social distancing measures, a national test, trace and isolate system is operational across the UK. The intensity and duration of the second wave in winter 2020/21 will be dependent on the effectiveness of these public health interventions, but will also be impacted by whether SARS-CoV-2 transmits more effectively in the winter than the summer. Mathematical models used to predict the transmission and impact of COVID-19 in the UK assume that the virus will produce an immune response that prevents reinfection in the short term ^3^.

Existing studies from outside the UK suggest that incidence of human coronaviruses in temperate climates is usually highest during winter, but spring and summer peaks and year-round circulation at varying levels have also been found ^4– 9^. There is minimal evidence regarding immunity and risk of repeat infection, but reinfection with common strains (HCoV OC43/229E) has been documented ^10, 11^ and reinfection with SARS-CoV appears to be theoretically plausible as it has been shown that antibody titres appear to decline over time, with estimates for duration of protection up to three years ^12^.

Flu Watch is a cohort study measuring the community incidence and transmission of several respiratory viruses in England ^13^. The study has the advantage of identifying mild cases of respiratory infection regardless of whether they lead to medical attendance and can therefore measure community incidence of infection over time and reinfection regardless of severity. We aimed to describe the community incidence and seasonal patterns of seasonal coronavirus strains, assess the frequency of reinfection with homologous and heterogeneous strains, and among participants with two confirmed HCoV infections, examine how likely we were to observe the number of homologous reinfections if participants had no immunity.

## Methods

### Study design and procedure

This study is based on analysis of data collected as part of the Flu Watch study, a prospective community cohort study of the transmission and burden of acute respiratory illness in UK households. The full study design and methodology has been described previously ^13^. Follow-up occurred across three consecutive winter seasons (Nov-Mar 2006–2007, 2007–2008, 2008–2009), the summer and winter waves of the 2009 H1N1 influenza pandemic (May-Sep 2009, Oct-Feb 2009–2010) and ‘post-pandemic’ winter season (Nov-Mar 2010–2011).

Demographic data were collected at the start of each season and in this analysis we used age, sex, geographical region, quintile of Index of Multiple Deprivation 2007 (a composite measure of the socioeconomic status of small neighbourhoods) ^14^. Throughout the season, participants were contacted weekly (via telephone or emailed online surveys) and asked to provide reports stating whether anyone in the household had experienced symptoms of acute respiratory illness. During all days of illness, participants were asked to report their symptoms and whether they took any time off work or study. In addition, we requested that all participants experiencing respiratory symptoms (including feeling feverish, headache, having muscle aches, cough, sore throat, runny nose, blocked nose, and sneezing) provide self-administered nasal swabs on the second day of illness. In the first season, participants received swabs via the post only when they reported illness (so swabs are likely to have arrived later than day two of illness) and swabbing began in late December 2006. In all subsequent seasons, participants received swabs at the beginning of follow up and we requested swabs for all illnesses regardless of when they occurred during follow up. Full details of sample handling and testing are provided elsewhere ^13, 15^. All swabs were tested for HCoV during the first four seasons, but only selected swabs were tested for HCoV in the pandemic and post-pandemic winter seasons. Table 1 summarises respiratory virus PCR testing across Flu Watch seasons.

**Table 1.** Respiratory virus PCR * testing on nasal swabs across Flu Watch seasons.

	Nov 2006 – Mar 2007	Nov 2007 – Mar 2008	Nov 2008 – Mar 2009	May 2009 – Sep 2009	Oct 2009 – Feb 2010	Nov 2010 – Mar 2011
HCoV **	✓	✓	✓	✓	partial	partial
Influenza A (H1N1)	✓	✓	✓	✓	✓	✓
Influenza A (H3N2)	✓	✓	✓	✓	✓	✓
Influenza A (H1N1pdm09)	n/a	n/a	n/a	✓	✓	✓
Influenza B	✓	✓	✓	✓	✓	✓
RSV ***	✓	✓	✓	✓	✓	✓
hMPV ****	✓	✓	✓	✓	✓	✓
Adenovirus	✓	✓	✓	✓	partial	partal
Parainfluenza virus	✓	✓	✓	✓	partial	partial
Rhinovirus	✓	✓	✓	✓	partial	partial

*Polymerase chain reaction **Human Coronavirus ***Respiratory syncytial virus ****Human metapneumovirus

We have published the full dataset used in this study (see underlying data).

### Participants

Participants were randomly selected from participating general practice lists in England. All household members of each participant were invited. Households were recruited before each winter season. From 2008–2009, households that had previously participated were also re-invited to the study. Participants were eligible if all household members agreed to participate for the full season and adult household members (aged 16 years and older) agreed to provide blood samples for influenza-related research. Participants were not eligible if their household was larger than 6 people, if any household member suffered from terminal or severe illness or incapacity, or had heavy concurrent involvement in other research.

### Outcomes

The primary outcome of interest in this study was PCR-confirmed coronavirus infection in participants. Three coronavirus strains were tested: HCoV-NL63, HCoV-OC43, and HCoV-229E.

### Statistical analysis

We calculated the rate of PCR-confirmed coronavirus infection per 100,000 person-weeks. Follow-up began at the start of each season and ended at the earliest of the final report of symptoms or the end of the season. We stratified rates by participants’ age, sex, geographical region, quintile of Index of Multiple Deprivation 2007 ^14^, and study season. We used a mixed poisson model to estimate rate ratios for confirmed HCoV. The dependent variable was the count of HCoV infections per season, the independent variables were participant characteristics at the start of each season plus an offset for the duration of follow-up, and the model was clustered by individual and household. For these descriptive analyses, we excluded the pandemic and post-pandemic winter seasons (2009–2010 and 2010–2011) as not all samples were tested for HCoV during these two seasons.

We used HCoV strains in participants with repeat infections to test the evidence for homologous immunity after infection. We started from a ‘null’ hypothesis of no immunity, and assumed that in this scenario the distribution of strains among participants with a second infection would be the same as in the entire cohort. If there is at least some immunity, we expected to see a pattern in which participants with a previous infection were less likely to have the same strain twice. For the eight participants with repeat infections, we created 100,000 simulations in which strain of the second infection (HCoV-NL63, HCoV-OC43, or HCoV-229E) was sampled with the probabilities observed in the entire cohort, and counted the number of homologous reinfections (zero to eight). We interpreted the proportion of simulations with as many or fewer homologous reinfections than in the observed data as evidence of immunity. In other words, the analysis attempts to examine the following question “is it likely that participants would have got more homologous reinfections if infection provided no immunity?” Figure S1 (extended data16 shows the first ten simulations and provides further detail of this method. For this analysis, we included data from the final two winter seasons (2009–2010 and 2010–2011).

We also estimated the percent of illnesses that were swabbed and tested for the relevant seasonal panel of viruses (see Table S1 extended data ^16^) as well as for HCoV by season to aid interpretation of results.

Analysis was conducted using R version 3.6.2.

### Ethics and consent

The ethical protocol for Flu Watch was approved by the Oxford MultiCentre Research Ethics Committee. (06/Q1604/103). Participants gave written informed consent (proxy consent for children).

## Results

Approximately 10% of invited households agreed to participate in Flu Watch. Compared to the national population, the study population underrepresented young adults; people living in socially deprived areas, north England, west Midlands, and London; and people of non-white ethnic origin. We included 51,002 person-weeks of follow-up and 2,907 person-seasons in the first four seasons.

A total of 1,104 swabbed illnesses were tested for HCoV and 199 cases were confirmed in the first four seasons. This total excludes six HCoV positive swabs (three in winter 2008–2009 and three in winter 2009–2010) as they were submitted without a participant ID. The percent of illnesses that were swabbed varied by season with lower adherence during Nov 2007 – Mar 2008 and May 2009 – Sept 2010 (61.5% and 57.0% respectively) and better adherence in the other seasons ranging from 83.6%-96.9% (see extended data 2 table S1). In the last two seasons when there was only partial testing (Oct 2009 – Feb 2010 and Nov 2010 – Mar 2011) the percent of illnesses swabbed and tested for HCoV was 14.0% and 24.5% respectively, which is why we did not report HCoV rates for these seasons.

We calculated an HCoV incidence rate of 390 per 100,000 person-weeks (95% CI 338–448) across the first four seasons. The maximum rate of HCoV was found in the 0–4 age group, with rate ratios lower in the 5–15; 45–64 and 65+ age groups compared to the 0–4 age group. Rates were similar in males and females, by geographical region, and by level of deprivation. Rates and rate ratios for participant characteristics are shown in Table 2.

**Table 2.** Baseline characteristics of study participants and HCoV PCR+ illness rates across the first four seasons (Nov-Mar 2006/7; Nov-Mar 2007/8; Nov-Mar 2008/9; May-Sep 2009).

Variable	Level	Individuals	Person- Seasons	Person- weeks	HCoV * PCR **+	HCoV PCR **+/100,000 person-weeks (95% CI)	Rate ratio (unadjusted)	Rate ratio (adjusted)
Total		1,847 (100.0%)	2,907	51,002	199	390 (338-448)		
Age group	0–4	111 (5.8%)	153	2,530	18	711 (422-1,124)	1	1
	5–15	272 (14.3%)	405	7,021	27	385 (253-560)	0.51 (0.27-0.96)	0.50 (0.26-0.94)
	16–44	537 (28.2%)	773	13,180	60	455 (347-586)	0.69 (0.39-1.20)	0.67 (0.38-1.17)
	45–64	650 (34.1%)	1,035	18,487	65	352 (271-448)	0.53 (0.30-0.94)	0.55 (0.31-0.97)
	65+	337 (17.7%)	541	9,784	29	296 (199-426)	0.45 (0.23-0.86)	0.48 (0.25-0.91)
Sex	Female	973 (52.7%)	1,543	26,986	105	389 (318-471)	1	1
	Male	874 (47.3%)	1,364	24,016	94	391 (316-479)	1.01 (0.76-1.34)	1.01 (0.76-1.34)
Region	East & East Midlands	318 (17.1%)	484	8,573	29	338 (227-486)	1	1
	London	116 (6.2%)	159	2,564	11	429 (214-768)	1.44 (0.65-3.18)	1.24 (0.56-2.76)
	North	273 (14.7%)	394	6,898	26	377 (246-552)	1.16 (0.63-2.14)	1.14 (0.61-2.12)
	South East	297 (16.0%)	479	8,179	22	269 (169-407)	0.85 (0.45-1.58)	0.83 (0.44-1.57)
	South West	698 (37.5%)	1,154	20,545	92	448 (361-549)	1.33 (0.82-2.17)	1.49 (0.91-2.44)
	West Midlands	159 (8.5%)	237	4,243	19	448 (270-699)	1.42 (0.73-2.78)	1.32 (0.67-2.59)
IMD *** 2007	1 - most deprived	99 (4.6%)	136	2,453	14	571 (312-958)	1	1
	2	284 (13.3%)	366	6,499	30	462 (311-659)	0.83 (0.38-1.81)	0.89 (0.41-1.95)
	3	534 (25.0%)	804	14,229	62	436 (334-559)	0.76 (0.37-1.55)	0.74 (0.36-1.53)
	4	513 (24.1%)	730	12,924	46	356 (261-475)	0.64 (0.31-1.33)	0.69 (0.33-1.44)
	5 - least deprived	409 (19.2%)	578	9,971	44	441 (321-592)	0.83 (0.39-1.73)	1.03 (0.49-2.16)
	Missing	293 (13.7%)	293	4,926	3	61 (13-178)	0.09 (0.02-0.33)	1.76 (0.30-10.21)
Season	Nov-Mar 2006/7	602 (20.7%)	602	10,751	42	391 (282-528)	1	1
	Nov-Mar 2007/8	779 (26.8%)	779	14,183	70	494 (385-624)	1.30 (0.85-1.99)	1.31 (0.85-2.02)
	Nov-Mar 2008/9	729 (25.1%)	729	12,315	83	674 (537-835)	1.68 (1.12-2.53)	1.68 (1.12-2.53)
	May-Sep 2009	797 (27.4%)	797	13,753	4	29 (8-74)	0.07 (0.03-0.21)	0.05 (0.01-0.21)
Month	Jan	1,737 (15.4%)	2,023	8,534	61	715 (547-918)		
	Feb	1,740 (15.5%)	2,033	8,040	61	759 (580-975)		
	Mar	1,722 (15.3%)	2,007	9,241	13	141 (75-241)		
	May ****	681 (6.0%)	681	2,643	0	0 (0-140)		
	Jun ****	679 (6.0%)	679	3,346	3	90 (18-262)		
	Jul ****	649 (5.8%)	649	2,514	0	0 (0-147)		
	Aug	602 (5.3%)	602	2,941	0	0 (0-125)		
	Sep	670 (6.0%)	670	2,309	1	43 (1-241)		
	Nov	1,114 (9.9%)	1,280	3,050	4	131 (36-336)		
	Dec	1,666 (14.8%)	1,942	8,384	56	668 (505-867)		

* Human Coronavirus ** Polymerase chain reaction ***Index of Multiple Deprivation **** *rates for May-Sep are based on data from 2009 alone*

**Figure 1.**
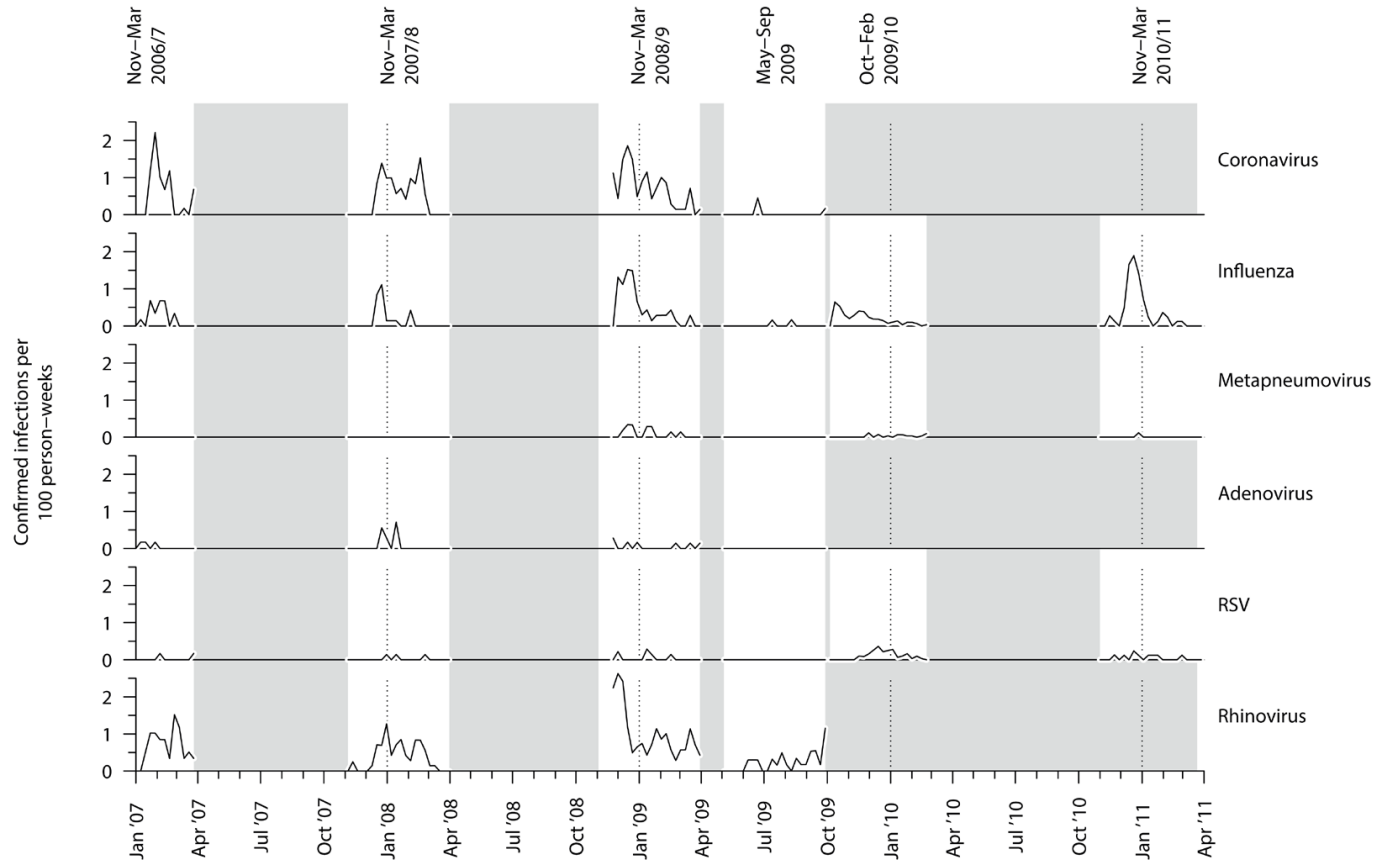
Weekly rates of PCR-confirmed viral respiratory diseases. PCR= Polymerase chain reaction, RSV = Respiratory syncytial virus.

Rates were higher in winter seasons than in the summer season of May-Sep 2009, during which four cases of HCoV were detected. Combining data across the first four seasons showed rates were highest in the month of February (759; 95%CI 580–975). Considering all respiratory viruses tested for, HCoV and influenza both peaked in winter and then declined, whereas hMPV, adenovirus, RSV, and rhinovirus showed no obvious winter peak, though this may relate to the small number of cases we detected ( Figure 1).

Of 216 participants with a first confirmed HCoV infection during any of the six seasons, eight had a second confirmed HCoV infection (all eight were from different households). These participants are shown in Table 3. None of the eight participants had the same strain twice. No participants had a third confirmed HCoV infection. Based on simulations assuming no immunity, the probability of zero homologous reinfections in these eight participants was 3.48%, suggesting some evidence for immunity (Figure S2 extended data ^16^).

**Table 3.** Participants with repeated confirmed coronavirus infections.

	First confirmed infection		Second confirmed infection		Weeks between infections
Number	Week commencing	Strain	Week commencing	Strain	
1	04-Feb-08	229E	19-Jan-09	NL63	50
2	24-Nov-08	NL63	21-Dec-09	229E	56
3	01-Dec-08	OC43	16-Mar-09	229E	15
4	15-Dec-08	OC43	02-Feb-09	NL63	7
5	22-Dec-08	NL63	09-Feb-09	OC43	7
6	22-Dec-08	OC43	09-Feb-09	NL63	7
7	12-Jan-09	NL63	22-Jun-09	229E	23
8	16-Feb-09	229E	21-Dec-09	OC43	44

## Discussion

Our study shows that HCoV appears to follow a seasonal pattern in England, with peaks occurring during winter seasons and broadly at the same time as influenza. We collected data during one summer season that coincided with the start of the 2009 H1N1 influenza pandemic, and during this period we found a small amount of ongoing HCoV transmission. Our results provide some evidence of immunity against homologous reinfection.

Our results support existing evidence for seasonality of HCoV in England with reduced transmission during summer months. To our knowledge, this is the first published study of HCoV seasonality in England and the first to show continued transmission during summer months. A 2010 review ^9^ of HCoV-NL63 found that it broadly followed a winter seasonal distribution in temperate climates (Belgium, Canada, France, Germany, Italy, Switzerland) with greater variation in tropical climates with China (Hong Kong) showing a spring and summer distribution (one study) and peaks in autumn in Thailand and October in Taiwan. Two further studies have been published since this review. The first was a community surveillance study in Utah, USA, which showed a broadly winter seasonal pattern ^8^. Another study of swab specimens from adults and children with fever and acute upper respiratory infection symptoms in Guangzhou, China, found transmission throughout the year with a peak in February ^7^.

Limited data exist on the immunising effect of previous infection with HCoV. Our data provide additional support for the immunising effect of infection in the short to medium term, but reinfection has been documented elsewhere. Our results should be interpreted with caution due to our sample size and the fact that we have not accounted for seasonal variations in strains, but it should also considered in the context of existing literature on immunity to HCoV, including community surveillance and experimental reinfection challenge studies. In a 1971 study of 937 medical students, reinfection with HCoV-229E was detected and infection with other respiratory viruses did not stimulate significant complement factor or neutralising antibody titre rises against HCoV-229E ^10^. A combined paediatric hospital inpatient and household community surveillance study conducted in Kenya found second infections with HCoV-NL63 (20.9%), HCoV-OC43 (5.7%), and HCoV-229E (4.0%) over the six years of the study. This study provided evidence to rule out genotype switching as a possible mechanism for reinfection. Two studies have also demonstrated experimental HCoV reinfection in humans ^17, 18^. At the time of writing, one animal study has been conducted to examine the possibility of SARS-CoV-2 reinfection ^19^. In this study, four 3- to 5-year old rhesus macaques were inoculated with SARS-CoV-2 and after the disappearance of symptoms, two were rechallenged and no viral load was detected. This study is important as it provides the only data we currently have on SARS-CoV-2, but it should also be interpreted with caution due to the fact that it is a primate study with a small sample size.

There are several additional limitations to our analysis and data. Our PCR data are reliant on participants sampling when symptomatic, which means that we will have not detected asymptomatic infection leading to underascertainment of such cases and as a result our estimates of rates will underestimate the true community burden. It is likely that we also received fewer samples from those who were minimally symptomatic. Our results therefore represent minimal burden estimates and we were unable to examine this further as we have no paired serological data on HCoV, although stored residual sera are available for this cohort and could be examined in future. Additionally, we were not able to calculate rates of confirmed HCoV infection in the final two winter seasons because not all swabs were tested for HCoV. Participants were advised to collect samples on day two of symptoms as Flu Watch was primarily set up to examine influenza and we are uncertain whether or not this is the optimum day for sampling those with HCoV. We only have one year of data collection during summer, during which time adherence to swabbing was lower than winter periods. Our ability to confidently estimate the levels of transmission during the summer is limited as a result of this, as is our description of seasonality, although as we have discussed earlier, these results are consistent with the wider literature for HCoV transmission in temperate climates. A conclusive picture about the seasonal pattern of SARS-CoV-2 will require monitoring over several years to confirm. The generalisability of our results to SARS-CoV-2 has uncertainties, but given the lack of data on this novel virus, we believe that these data can help inform the public health response. At this stage in the pandemic, it appears to be the case that the clinical features of mild cases of SARS-CoV-2 are similar to NL63, OC43 and 229E, but the likelihood of developing severe disease or dying is much higher although considerably less than in SARS-CoV and MERS-CoV ^20, 21^. Finally, because of small numbers in sub-groups it has not been possible to stratify our results socio-economic status or ethnicity despite the fact that they are increasingly recognised as important factors associated with COVID-19 outcomes.

In summary, our data provide additional support for a winter seasonal pattern of HCoV in the UK and that infection has an immunising effect against subsequent reinfection in the short to medium term. For COVID-19, in the context of intensive control measures it may prove difficult to assess the extent to which virus transmission is impeded by summer conditions. Comparing transmission the patterns in northern and southern hemispheres (where seasons are reversed) will be of help in providing early data on this. We also need further research to assess the strength and duration of immune protection following COVID-19 exposure. Whilst our results can help inform the response and modelling to SARS-CoV-2 in the UK, there are important research questions that need answering from community surveillance studies that are relevant to the policy and public health response. We urgently need to know the true extent of community transmission, including estimates of the asymptomatic fraction of SARS-CoV-2, the symptomatology in community cases and case hospitalisation rates in confirmed cases and how this varies over time and season. Additionally, we need to know what the duration of viral shedding is and whether there is evidence for repeat infection of SARS-CoV-2 in humans.

## Data availability

### Underlying data

UCL Discovery: Dataset: Seasonality and immunity to laboratory-confirmed seasonal coronaviruses (HCoV-NL63, HCoV-OC43, and HCoV-229E): results from the Flu Watch cohort study. https://doi.org/10.14324/000.ds.10093909 ^16^

This project contains the following underlying data:

- Aldridge_cov_seasonality_immunity_public_data_23_march_2020.csv (Flu Watch HCoV data)

### Extended data

UCL Discovery: Dataset: Seasonality and immunity to laboratory-confirmed seasonal coronaviruses (HCoV-NL63, HCoV-OC43, and HCoV-229E): results from the Flu Watch cohort study. https://doi.org/10.14324/000.ds.10093909 ^16^

This project contains the following extended data:

- Aldridge_cov_seasonality_immunity_public_code_revised.R (Analysis replication code)
- Aldridge_Extended data 1.pdf (Pdf file containing Table S1 and Figure S1 and S2)
  - Table S1. Illnesses swabbed and tested for HCoV by season
  - Figure S1: First ten simulations to evaluate evidence of homologous immunity
  - Figure S2: Probability of number of homologous reinfections in 8 participants, with the assumption of no immunity

Data are available under the terms of the Creative Commons Attribution 4.0 International license (CC-BY 4.0).
