# Supplementary material for: Seasonality and immunity to laboratory-confirmed seasonal coronaviruses (HCoV-NL63, HCoV-OC43, and HCoV-229E): results from the Flu Watch cohort study
Source: Wellcome Open Res. 2020 Dec 10;5:52. Originally published 2020 Mar 30. [Version 2] doi: 10.12688/wellcomeopenres.15812.2 (PMC7786426; doi:10.12688/wellcomeopenres.15812.2)
Supplement: Peer review history

# Peer review: Reviewer response for version 2

**DOI:** 10.21956/wellcomeopenres.18155.r41796

**Published:** 2021-01-05

**Authors:** David J. Muscatello

**Affiliations:** 1 School of Public Health and Community Medicine, University of New South Wales, Sydney, Australia

I have no further comments to make. Thank you for addressing my concerns.

Is the work clearly and accurately presented and does it cite the current literature?

Partly

If applicable, is the statistical analysis and its interpretation appropriate?

Yes

Are all the source data underlying the results available to ensure full reproducibility?

Yes

Is the study design appropriate and is the work technically sound?

Partly

Are the conclusions drawn adequately supported by the results?

Partly

Are sufficient details of methods and analysis provided to allow replication by others?

Yes

Reviewer Expertise:

Epidemiology, influenza, time series analysis, infectious diseases.

I confirm that I have read this submission and believe that I have an appropriate level of expertise to confirm that it is of an acceptable scientific standard.

# Peer review: Reviewer response for version 2

**DOI:** 10.21956/wellcomeopenres.18155.r41797

**Published:** 2020-12-10

**Authors:** Tom Wingfield

**Affiliations:** 1 Departments of Clinical Sciences and International Public Health, Liverpool School of Tropical Medicine, Liverpool, UK

The authors have responded fully to my comments and I am delighted to accept the current version of this article for indexing on Wellcome Open Research.

Is the work clearly and accurately presented and does it cite the current literature?

Yes

If applicable, is the statistical analysis and its interpretation appropriate?

Yes

Are all the source data underlying the results available to ensure full reproducibility?

Yes

Is the study design appropriate and is the work technically sound?

Yes

Are the conclusions drawn adequately supported by the results?

Yes

Are sufficient details of methods and analysis provided to allow replication by others?

Yes

Reviewer Expertise:

Clinical infectious diseases (HIV/TB), epidemiology, social determinants and consequences of ill-health.

I confirm that I have read this submission and believe that I have an appropriate level of expertise to confirm that it is of an acceptable scientific standard.

# Peer review: Reviewer response for version 1

**DOI:** 10.21956/wellcomeopenres.17340.r38795

**Published:** 2020-06-10

**Authors:** David J. Muscatello

**Affiliations:** 1 School of Public Health and Community Medicine, University of New South Wales, Sydney, Australia

In the context of the emerging COVID-19 pandemic, this article attempts to address two important questions - seasonality of human coronavirus infections in a temperate setting in the UK and the probability of re-infection by the same coronavirus in the time frame of a few years.

 A positive of the FluWatch study is that it examined infection incidence in a more-or-less representative sample of the population. This is a very rare occurrence in infection incidence study designs.

 The main limitation of the study is the limited number of non-winter seasons included - only one. A second limitation is that re-infection may have occurred during the periods during which follow-up did not occur - that is, the other missing summer seasons.

 I have a few minor concerns:

- spelling of influenza should be lower case throughout - it is not a proper noun.
- Human Metapneumovirus should be spelled with a lower case m for the same reason - see footnote of Table 1.
- Abstract - I would prefer to see "100,000 person weeks" included next to all of the rate statistics in the result section. The highest rate is in February - should be clear that the highest average monthly rate was in Feb because I assume this was an average for Feb over the three seasons that included Feb.
- Background 2nd paragraph - could also reference this study[ref-1].
- Would be helpful if methods included the specific respiratory symptoms that triggered follow-up from the original study.
- ​​Simulation description in methods could be explained better here and in the supplement. If I understand correctly, the aim was to compare the observed number of second homologous infections with the predicted number of second homologous infections. The predicted number was determined from 100,000 simulations that assumed that occurrence of a second homologous infection followed a distribution based on a null hypothesis that the probability of a second homologous infection was the same as the probability of the homologous strain occurring among all participants throughout the study period included. This simulation approach assumed that there was no residual immunity from a first infection. Thus, if the occurrence of the second homologous infection had a lower incidence than predicted then we might conclude that there is immunity to a second homologous infection. It's also not really clear why simulation was necessary when the simulation simply reproduces the distribution of infections observed in the study.
- The statement "The maximum rate of HCoV according to age was bimodal, peaking at ages 0–4 and ages 16–44" seems a bit of a stretch. All other age groups apart from 16-44 were lower than 0-4 based on adjusted RR, but 16-44 was not that much higher than all non-0-4 age groups.
- The results for monthly incidence are misleading e.g. in Table 2. May-Sep results are based on one season only. Remaining months based on three seasons. Are remaining months averages over 3 seasons? A footnote to the table could explain these issues.
- The authors have done well in stating that their results should be interpreted with caution. They could state more clearly that one important reason for this is that the summer results are based on one season only, and that's really insufficient to draw conclusions about seasonality which requires monitoring over several years to determine a seasonal pattern.

Is the work clearly and accurately presented and does it cite the current literature?

Partly

If applicable, is the statistical analysis and its interpretation appropriate?

Yes

Are all the source data underlying the results available to ensure full reproducibility?

Yes

Is the study design appropriate and is the work technically sound?

Partly

Are the conclusions drawn adequately supported by the results?

Partly

Are sufficient details of methods and analysis provided to allow replication by others?

Yes

Reviewer Expertise:

Epidemiology, influenza, time series analysis, infectious diseases.

I confirm that I have read this submission and believe that I have an appropriate level of expertise to confirm that it is of an acceptable scientific standard, however I have significant reservations, as outlined above.

# Response

**Published:** 2020-11-30

**Authors:** Robert Aldridge

**Affiliations:** UCL, UK

Thanks very much for this helpful review - we appreciate the time you have taken to carefully read and provide constructive feedback on the article. We have tried to address all the points you have raised and below we include your comments alongside in our responses in bold italics. We have numbered each of our responses in order that we can cross-reference them across all of the reviews to this article. 

**Reviewer comment 4:**

 Spelling of influenza should be lower case throughout - it is not a proper noun.

**Author response 4:**

 We have changed influenza to lower case throughout. 

**Reviewer comment 5:**

 Human Metapneumovirus should be spelled with a lower case for the same reason - see footnote of Table 1.

**Author response 5:**

 We have changed Human Metapneumovirus in Table 1 footnote. 

**Reviewer comment 6:**

 Abstract - I would prefer to see "100,000 person weeks" included next to all of the rate statistics in the result section. The highest rate is in February - should be clear that the highest average monthly rate was in Feb because I assume this was an average for Feb over the three seasons that included Feb.

**Author response 6:**

 We have added per 100,000 person-weeks to all rates in the abstract. The rate for the month Feb is not an average but is a rate calculated across all seasons using all cases in Feb as the numerator and person time at risk during Feb as the denominator.

**Reviewer comment 7:**

 Background 2nd paragraph - could also reference this study[ref-1].

**Author response 7:**

 [ref-1] did not appear to come through in the peer review response. 

**Reviewer comment 8:**

 Would be helpful if methods included the specific respiratory symptoms that triggered follow-up from the original study.

**Author response 8:**

 We have added the following text to the methods section:

 “In addition, we requested that all participants experiencing respiratory symptoms (including feeling feverish, headache, having muscle aches, cough, sore throat, runny nose, blocked nose, and sneezing) provide self-administered nasal swabs on the second day of illness.”

**Reviewer comment 9:**

 ​​Simulation description in methods could be explained better here and in the supplement. If I understand correctly, the aim was to compare the observed number of second homologous infections with the predicted number of second homologous infections. The predicted number was determined from 100,000 simulations that assumed that occurrence of a second homologous infection followed a distribution based on a null hypothesis that the probability of a second homologous infection was the same as the probability of the homologous strain occurring among all participants throughout the study period included. This simulation approach assumed that there was no residual immunity from a first infection. Thus, if the occurrence of the second homologous infection had a lower incidence than predicted then we might conclude that there is immunity to a second homologous infection. It's also not really clear why simulation was necessary when the simulation simply reproduces the distribution of infections observed in the study.

**Author response 9:**

 In response to this comment, we have reviewed and edited our description of our approach to this part of the analysis in the methods section, to hopefully clarify our intention with this analysis:

 “We used HCoV strains in participants with repeat infections to test the evidence for homologous immunity after infection. We started from a ‘null’ hypothesis of no immunity, and assumed that in this scenario the distribution of strains among participants with a second infection would be the same as in the entire cohort. If there is at least some immunity, we expected to see a pattern in which participants with a previous infection were less likely to have the same strain twice. For the eight participants with repeat infections, we created 100,000 simulations in which strain of the second infection (HCoV-NL63, HCoV-OC43, or HCoV-229E) was sampled with the probabilities observed in the entire cohort, and counted the number of homologous reinfections (zero to eight). We interpreted the proportion of simulations with as many or fewer homologous reinfections than in the observed data as evidence of immunity. In other words, the analysis attempts to examine the following question “is it likely that participants would have got more homologous reinfections if infection provided no immunity?”  Figure S1 (extended data 16) shows the first ten simulations and provides further detail of this method. For this analysis, we included data from the final two winter seasons (2009–2010 and 2010–2011).”

**Reviewer comment 10:**

****

 The statement "The maximum rate of HCoV according to age was bimodal, peaking at ages 0–4 and ages 16–44" seems a bit of a stretch. All other age groups apart from 16-44 were lower than 0-4 based on adjusted RR, but 16-44 was not that much higher than all non-0-4 age groups.

**Author response 10:**

 We explored this issue further as per Author response 2 to Reviewer 1, but do not feel additional analysis is possible. As a result we have edited our description of these results in response to this reviewer comment 10 to:

 “The maximum rate of HCoV was found in the 0–4 age group, with rate ratios lower in the 5-15; 45-64 and 65+ age groups compared to the 0–4 age group.”

**Reviewer comment 11:**

 The results for monthly incidence are misleading e.g. in Table 2. May-Sep results are based on one season only. Remaining months based on three seasons. Are remaining months averages over 3 seasons? A footnote to the table could explain these issues.

**Author response 11:**

 In Table 2 we have calculated the rate of HCoV PCR+ cases per 100,000 person-weeks. For each month we calculate this rate using the numerator as HCoV PCR+ cases and the denominator of Person-weeks across the whole month. The numerator and denominator for each month are each presented in Table 2 with the columns labelled, and the rate is specified as HCoV PCR**+/100,000 person-weeks

 (95% CI) in the column header. It is correct that May-Sep results are based on one season only and remaining months based on all seasons. 

 In the discussion we previously noted that “We only have one year of data collection during summer, during which time adherence to swabbing was lower than winter periods. Our ability to confidently estimate the levels of transmission during the summer is limited as a result of this, as is our description of seasonality, although as we have discussed earlier, these results are consistent with the wider literature for HCoV transmission in temperate climates.”

 In response to this reviewer comment 11 we have now added a footnote to Table 2 stating that: “****rates for May-Sep are based on data from 2009 alone”.

**Reviewer comment 12:**

****

 The authors have done well in stating that their results should be interpreted with caution. They could state more clearly that one important reason for this is that the summer results are based on one season only, and that's really insufficient to draw conclusions about seasonality which requires monitoring over several years to determine a seasonal pattern.

**Author response 12:**

 In addition to the following text in the discussion: 

 “We only have one year of data collection during summer, during which time adherence to swabbing was lower than winter periods. Our ability to confidently estimate the levels of transmission during the summer is limited as a result of this, as is our description of seasonality, although as we have discussed earlier, these results are consistent with the wider literature for HCoV transmission in temperate climates.”

 We have added the following text in response to this point:

 “A conclusive picture about the seasonal pattern of SARS-CoV-2 will require monitoring over several years to confirm.”

# Peer review: Reviewer response for version 1

**DOI:** 10.21956/wellcomeopenres.17340.r38640

**Published:** 2020-05-06

**Authors:** Tom Wingfield

**Affiliations:** 1 Departments of Clinical Sciences and International Public Health, Liverpool School of Tropical Medicine, Liverpool, UK

This is a well-written and executed article by a team that is well experienced in the field and have a strong track record.

 The findings are useful and highly topical amid the SARS-CoV-2 pandemic, especially with relation to the issue of immunity and re-infection.

 I have a few minor comments:

1. It would be great to see some of the data disaggregated by IMD and also ethnicity (or if not individual ethnicity perhaps proportion of BAME people in LSOA from where swab sent) as it is mentioned that these data are collected. This is highly pertinent to the evidence that is gathering about the differential impact of Covid-19 in BAME and amongst people of lower socioeconomic status. The issue of under-reporting in these groups is mentioned early in the manuscript and should also perhaps be mentioned in the limitations section.
2. Please can the authors comment/interpret the findings across age groups that are shown in Table 2 (lower HCoV PCR+ illness in older age groups compared to 0-4) and how these might be applied to help our understanding of SARS-CoV-2 transmission and illness, especially in light of potential lifting of lockdown measures including school closures.
3. Minor update to Covid-19 global/UK numbers will be required on re-submission of final version.

Congratulations to the authors on an interesting, important, topical, and highly readable piece. Thank you for considering me to review.

Is the work clearly and accurately presented and does it cite the current literature?

Yes

If applicable, is the statistical analysis and its interpretation appropriate?

Yes

Are all the source data underlying the results available to ensure full reproducibility?

Yes

Is the study design appropriate and is the work technically sound?

Yes

Are the conclusions drawn adequately supported by the results?

Yes

Are sufficient details of methods and analysis provided to allow replication by others?

Yes

Reviewer Expertise:

Clinical infectious diseases (HIV/TB), epidemiology, social determinants and consequences of ill-health.

I confirm that I have read this submission and believe that I have an appropriate level of expertise to confirm that it is of an acceptable scientific standard, however I have significant reservations, as outlined above.

# Response

**Published:** 2020-11-30

**Authors:** Robert Aldridge

**Affiliations:** UCL, UK

Thanks very much for this helpful review - we appreciate the time you have taken to carefully read and provide constructive feedback on the article. We have tried to address all the points you have raised and below we include your comments alongside in our responses in bold italics. We have numbered each of our responses in order that we can cross-reference them across all of the reviews to this article. 

**Reviewer comment 1:**

 It would be great to see some of the data disaggregated by IMD and also ethnicity (or if not individual ethnicity perhaps proportion of BAME people in LSOA from where swab sent) as it is mentioned that these data are collected. This is highly pertinent to the evidence that is gathering about the differential impact of Covid-19 in BAME and amongst people of lower socioeconomic status. The issue of under-reporting in these groups is mentioned early in the manuscript and should also perhaps be mentioned in the limitations section.

**Author response 1:**

 Thank you for these suggestions which we have considered carefully. Table 2 provides data by IMD, but unfortunately it has not been possible to stratify other results of the study (e.g. Figure 1 or Table 3) by ethnicity status or IMD due to low number of events in these groups. We have added the following note about this in the discussion:

 “Finally, because of small numbers in sub-groups it has not been possible to stratify our results socio-economic status or ethnicity despite the fact that they are increasingly recognised as important factors associated with COVID-19 outcomes.”

**Reviewer comment 2:**

 Please can the authors comment/interpret the findings across age groups that are shown in Table 2 (lower HCoV PCR+ illness in older age groups compared to 0-4) and how these might be applied to help our understanding of SARS-CoV-2 transmission and illness, especially in light of potential lifting of lockdown measures including school closures.

**Author response 2:**

 Table 2 suggests that compared to the 0-4 age group, all other ages have a lower rate ratio of HCoV PCR+ illness. We are very cautious in our interpretation of this suggestion as the 0-4 age group also had the smallest number of events. In response to this reviewer comment we explored the possibility of modelling age as a continuous variable to improve our modelling approach. We also explored the possibility of including age with polynomials/splines. Unfortunately both of these approaches resulted in models that were more difficult to interpret and did not help with interpreting differences in HCoV PCR+ illness in older age groups compared to 0-4 as suggested. As a result, we do not think we can explore this issue further. However, we have edited the text describing these results as set out in response to Reviewer 2 comment 10. 

**Reviewer comment 3:**

 Minor update to Covid-19 global/UK numbers will be required on re-submission of final version.

**Author response 3:**

 We have updated our analyses with the latest Covid-19 global/UK numbers and edited the text in the introduction in light of the current situation.
